# Maternal and foetal factors contributing to caesarean section rates at Chris Hani Baragwanath Academic Hospital, a tertiary care centre in South Africa

**DOI:** 10.4102/jcmsa.v4i1.325

**Published:** 2026-07-10

**Authors:** Linda C. Taptue Noumsi, Evelyn Ngwa Lumngwena, Poovangela Naidoo

**Affiliations:** 1 Department of Obstetrics and Gynaecology, School of Clinical Medicine, Faculty of Health Sciences, University of the Witwatersrand, Johannesburg, South Africa; 2 School of Clinical Medicine, Faculty of Health Sciences, University of the Witwatersrand, Johannesburg, South Africa; 3 Centre for the Study of Emerging and Re-emerging Pathogens, Institute of Medical Research and Medicinal Plant Studies, Ministry of Scientific Research and Innovation, Yaoundé, Cameroon

**Keywords:** caesarean section, Robson classification, caesarean indications, near-miss, foetal outcome

## Abstract

**Background:**

Caesarean section (CS) rates remain high globally, increasing healthcare costs and maternal-neonatal complications. The World Health Organization (WHO) recommends the Robson Ten Group Classification System (RTGCS) to assess and compare CS rates and reduce primary CS, which contributes to repeat procedures and associated risks. This study aimed to analyse indications for CS using RTGCS, maternal outcomes using WHO near-miss criteria, and pre- and post-delivery foetal conditions at a tertiary referral hospital.

**Methods:**

A retrospective cross-sectional study was conducted on CS deliveries in an African hospital from 01–30 June 2022. Data were analysed using bivariate and multivariate analysis with Fisher’s exact test.

**Results:**

Of the 1,306 deliveries during the study period, 695 (53.2%) were CS. Robson Group 5 was the largest contributor (32.2%), followed by Groups 2 (16.4%) and 4 (12.8%). Foetal distress was the leading indication for CS (47.2%), followed by previous CS (declining vaginal birth after caesarean [VBAC]) (8.2%) and previous uterine scar × 2 in labour (5.8%). Mothers with a previous uterine scar accounted for 25.1% of all CSs, with 6.9% of them requiring high care. Maternal near-miss events occurred in 0.6% of cases. Group 5 had the highest maternal morbidity. Maternal risk factors that contributed to poor foetal outcome in this category were previous CS, chronic hypertension, gestational diabetes, poor obstetric history and obesity. Preoperatively, 62.4 % of foetuses had pathological non-stress tests (NSTs); 30.7% of NSTs were normal. Despite this, neonatal outcomes were favourable. Although no umbilical cord pH was done when the Apgar was > 7, those babies were discharged and coped well in the ward. The stillbirth risk was 12 times higher (OR 12.10, CI 4.29–34.14, *p* < 0.001) in women with adverse maternal outcomes, and low 5-min Apgar scores had a 6-fold increased odds in these women (OR 5.83, CI 2.79–12.18, *p* < 0.001). Maternal near-miss events were strongly associated with adverse neonatal outcomes; the main causes of maternal near misses were peripartum haemorrhage and sepsis. Women who experienced a near-miss had 12 times the odds of stillbirth (OR 12.10, 95% CI 4.29 –34.14, *p* < 0.001) and nearly six times the odds of a low Apgar score at 5 min (OR 5.83, 95% CI 2.79–12.18, *p* < 0.001).

**Conclusion:**

High CS rates reflect tertiary care burden, with the majority falling within Robson Group 5. This group was the most frequent contributor to CS, and foetal distress was the leading indication for CS.

**Contribution:**

This was followed by factors related to previous uterine scars. Women with prior uterine scars frequently had a repeat CS and showed notable care needs. Although most foetuses had abnormal preoperative NSTs, overall neonatal outcomes were favourable. However, maternal near miss events, though rare, were strongly associated with significantly increased risks of stillbirth and poor neonatal outcomes. Strategies are needed to prevent primary CS, thus reducing maternal and perinatal morbidity.

## Introduction

There is a global concern over the increasing caesarean section (CS) rate because of the increased costs, burden on the health system, and associated maternal and neonatal complications.^1^ In South Africa, the national CS rate was 32.3% in 2022, which rose from 28% in 2020.^2^ This was accompanied by a case fatality rate of 122.3 per 100 000 deliveries and a reduced incidence of perioperative haemorrhage to 14.4% from 22.3% previously.^3^ Preventing the first CS is critical, as it significantly increases the risk of repeat CS and related complications. Tertiary hospitals typically report higher CS rates owing to high-risk referrals.^4^

While the South African institutional maternal mortality rate (iMMR) decreased to 98.8 per 100 000 live births in 2017–2019,^5^ the corrected MMR rose to 126.1 per 100 000 live births in 2020, a 26% increase, coinciding with a CS rate of 28.1% in the public sector.^6^ Mortality risks associated with CS remain three times higher than with vaginal delivery, with previous CS being a key risk factor for obstetric haemorrhage.^7^

Neonatal outcomes are also affected by CS. Guidozzi et al. found that 11.6% of neonates born via CS required high-care admission compared with 6.6% via vaginal delivery (OR 1.865, *p* = 0.001).^8^ Emergency CSs showed a significantly higher perinatal mortality, particularly during advanced labour stages.^9^

To address rising CS rates and to guide CS reduction strategies, the World Health Organization (WHO) recommends using the Robson Ten-Group Classification System (TGCS) for standardised CS audits (Table 1).^10^ This tool has subsequently been used in numerous studies with varying results; for example, a retrospective study (2014–2018) at a hospital in Pretoria that used Robson’s classification for CSs found that maternal near-miss cases accounted for 69.6% of the caesarean rate.^12^ Meanwhile, a meta-analysis of 44 studies by Radaf et al’s showed Robson Group 5 rates ranged from 51.2% in Northern Europe to 95.0% in Southern Europe.^13^ This study used the Robson classification to evaluate the indications for CS and to assess maternal and foetal outcomes using WHO near-miss criteria in a tertiary hospital setting in Johannesburg, South Africa, given the high CS rate in the country.

**TABLE 1:** Robson’s Ten Group Classification System, including sub-groups.

Group	Description
1	Nulliparous, singleton, cephalic, term births without previous caesarean section in spontaneous labour
2	Nulliparous, singleton, cephalic, term births without previous caesarean section with induced labour or prelabour caesarean section
3	Multiparous, singleton, cephalic, term births without previous caesarean section in spontaneous labour
4	Multiparous, singleton, cephalic, term births without previous caesarean section with induced labour or prelabour caesarean section
5	Previous caesarean section, singleton, cephalic, term births
6	Nulliparous singleton breech births
7	Multiparous singleton breech births, including previous caesarean section
8	Multiple pregnancies, including a previous caesarean section
9	Transverse and oblique lie, including a previous caesarean section
10	Preterm (< 37 weeks), singleton, cephalic births, including previous caesarean section

*Source:* Tiwari M, Singh S, Kushwah BS. Robson’s ten group classification: a tool for predicting caesarean section rates. Int J Reprod Contracept Obstet Gynecol. 2023;12(3):595–602^11^

## Research methods and design

This was a retrospective cross-sectional analysis of the medical records of all women who delivered by CS at Chris Hani Baragwanath Academic Hospital (CHBAH), a tertiary care and referral hospital, from 01–30 June 2022. The maternity ward comprises approximately 300 beds, and the majority of patients are admitted from the hospital antenatal clinic (ANC) or referred to from other surrounding hospitals. The hospital has approximately 17 000 deliveries annually, with approximately 8000 CSs performed annually. At the last census, the hospital served a population of 1.5 million people and was the only tertiary hospital in Soweto.

Women included in the study were traced from the theatre registers, and relevant data were extracted from the maternity records.

Data were captured using the REDCap database (Vanderbilt University Medical Center, Nashville, Tennessee, United States) and analysed descriptively; means (and standard deviations) for parametric continuous data, or medians (and interquartile ranges [IQRs]) for non-parametric data, using Stata version 15.0 (StataCorp, College Station, Texas, United States).

Fischer’s exact test was used to determine significant differences in the proportions of various indications for CS and to assess its effect on maternal outcomes according to the WHO near-miss criteria across the 10 Robson classification groups.

### Ethical considerations

Ethical approval to conduct this study was obtained from the Human Research Ethics Committee of the University of the Witwatersrand, Johannesburg (No. M221009). Ethics clearance was sought, and permission was obtained from all administrative authorities prior to the study’s data collection. A waiver of individual participant informed consent was requested from the ethics committee given the retrospective nature of the study, and pseudonymisation was employed to protect participants’ confidentiality during analyses and the publication of results.

## Results

### Overall deliveries and maternal characteristics

Of 1306 deliveries in June 2022, 695 were CS, yielding a CS rate of 53.2%. Most women (80.4%) booked antenatal care before 20 weeks, with 52.9% having an early ultrasound. Most referrals were from Lenasia Extension Clinic (10.6%), Stretford (4.6%) and Chiawelo (4.5%). The majority were singleton pregnancies, 674 (97%), and most were delivered at term, 529 (76.1%). Full demographic and antenatal characteristics are presented in Table 2.

**TABLE 2:** Summary of maternal demographic, antenatal and clinical characteristics at the time of delivery (*N* = 695).

Description	Categories	*n*	%	Median	Range
Number of visits	< 4 visits	145	20.9	-	-
	4–8 visits	427	61.4	-	-
	> 10 visits	102	14.7	-	-
	None and unbooked	21	3.0	-	-
Age (years)	-	-	-	29	24–34
BMI	< 18 (Underweight)	6	0.9	-	-
	18–24.9 (Normal)	175	25.9	-	-
	25–29.9 (Overweight)	188	27.8	-	-
	30–34.9 (Class 1 Obesity)	143	21.2	-	-
	35–39.9 (Class 2 Obesity)	84	12.4	-	-
	≥ 40 (Class 3 Obesity)	80	11.8	-	-
MUAC (cm)	-	-	-	29	26–33
Booking status	Early	560	80.7	-	-
	Late	114	16.4	-	-
	Unbooked	21	3.2	-	-
Parity	0	219	31.4	-	-
	1	179	25.8	-	-
	2	185	26.6	-	-
	3	85	12.2	-	-
	4	22	3.2	-	-
	5	5	0.7	-	-
Gravidity	1	188	27.1	-	-
	2	171	24.6	-	-
	3	176	25.3	-	-
	4	102	14.7	-	-
	5	35	5.0	-	-
	6	19	2.7	-	-
	7+	4	0.6	-	-
Number of foetuses	Singleton	674	97.0	-	-
	DCDA twins set	15	2.2	-	-
	MCDA twins set	5	0.7	-	-
	Unknown chronicity	1	0.1	-	-
RPR status	Positive	13	1.9	-	-
Rh status	Negative	14	2.0	-	-
HIV status	Positive	168	24.2	-	-
CD4 count	-	-	-	516	312–646
EGA	< 36 + 6 d	166	23.9	-	-
	37–40 + 6 d	436	62.7	-	-
	> 41/40	93	13.4	-	-
Placenta location	Normal	689	99.1	-	-
	Previa	3	0.4	-	-
	Low lying	3	0.4	-	-
Presentation	Cephalic	657	94.5	-	-
	Breech	34	4.9	-	-
	Shoulder	2	0.3	-	-
	Compound	2	0.3	-	-
Mode of delivery	Emergency CS	615	88.5	-	-
	Elective CS	80	11.5	-	-
Induction of labour	No	482	69.4	-	-
	Yes	131	18.8	-	-

EGA, estimated gestational age; RH, rhesus status; BMI, body mass index; MUAC, middle upper arm circumference; RPR, rapid plasma reagent; CS, caesarean section; MCDA, monochorionic diamniotic; DCDA, dichorionic diamniotic.

### Indications for caesarean section

Emergency CS accounted for 88.5% of all CSs. The most common indication was foetal distress, 328 (47.2%), followed by previous CS declining vaginal birth after caesarean (VBAC) 57 (8.2%), previous CS (×2) in labour 40 (5.8%), and elective repeat CS (×2) 27 (3.9%). Failed VBAC, failed induction, and abruptio placenta each contributed 3%, 3% and 2.9%, respectively (Table 3).

**TABLE 3:** Primary indications for caesarean sections among women delivering at Chris Hani Baragwanath Academic Hospital during the study period (*N* = 695).

Indication for caesarean section	*n*	%
Foetal distress	328	47.2
Declined VBAC	57	8.2
Prev caesarean × 2 in labour	40	5.8
Prev caesarean section × 2 elective	27	3.9
Failed VBAC	21	3.0
Failed IOL	21	3.0
Abruptio placenta	20	2.9
Multiple gestation	19	2.8
Cephalopelvic disproportion	18	2.6
Previous caesarean section × 1 and hypertensive-related disorders	17	2.5
Breech	16	2.3
Severe pre-eclampsia	15	2.2
Poor progress of labour	13	1.9
Eclampsia	7	1.0
Previous caesarean section × 3	6	0.9

VBAC, vaginal birth after caesarean section; IOL, induction of labour.

The Robson classification identified Group 5 (previous CS, singleton, cephalic, ≥ 37 weeks) was the largest contributor to the CS rate, 224 (32.2), followed by Group 2 (nulliparous, induced or pre-labour CS, 114 [16.5%]) and Group 4 (multiparous, induced or pre-labour CS, 89 [12.8%]) (Table 4).

**TABLE 4:** Distribution of caesarean section indications stratified by Robson classification group.

Indication	Robson group																				*p*-value
	1		2		3		4		5		6		7		8		9		10		
	*n*	%	*n*	%	*n*	%	*n*	%	*n*	%	*n*	%	*n*	%	*n*	%	*n*	%	*n*	%	
Foetal distress	62	73.8	87	73.7	35	67.30	63*	70.8	44	19.6	-	-	-		4	16.0	1	25.0	32	37.6	< 0.001*
CPD	10	10.7	3	2.6	3	5.80	2	2.2	-	-	-	-	-	-	-	-	-	-	-	-	< 0.001*
Failed IOL	-	-	10	8.8	1	1.90	6	6.7	-	-	-	-	-	-	-	-	-	-	4	4.7	< 0.001*
Postdate + Failed IOL	-	-	1	0.9	-	-	-	-	-	-	-	-	-	-	-	-	-	-	-	-	< 0.001*
Failed AOL	2	2.4	1	0.9	1	1.90	3	3.4	-		-	-	-	-	-	-	-	-	-	-	-
Twins leading breech	-	-	-	-	-	-	-	-	2	0.9	-	-	-	-	11	44.0	-	-	-	-	< 0.001*
Twins very preterm	-	-	-	-	-	-	-	-	-	-	-		1	7.7	5	20.0	-	-	-	-	< 0.001*
Breech presentation	-	-	-	-	-	-	-	-	-	-	5	100	11	84.6	-	-	-	-	-	-	< 0.001*
Eclampsia	1	1.2	-	-	-	-	-	-	1	0.4	-	-	-	-	-	-	1	25	4	4.7	< 0.001*
Severe pre-eclampsia	1	1.1	1	0.9	-	-	1	1.1	3	1.3	-		-	-	2	8.0	1	25	7	8.2	< 0.001*
HELLP syndrome	-	-	-	-	-	-	-	-	-	-	-	-	-	-	1	4.0	-	-	6	7.0	-
Abruptio placenta	2	2.3	2	1.7	-	-	2	2.2	2	0.9	-	-	-	-	-	-	-	-	12	14.2	0.396*
APH unknown origin	-	-	-	-	1	1.90	1	1.1	1	0.4	-	-	-	-	1	4.0	1	25	3	3.5	-
Placenta praevia	-	-	-	-	-	-	-	-	3	0.4	-	-	-	-	-	-	-	-	-	-	-
Macrosomia	-	-	1	0.9	-	-	-	-	2	0.9	-	-	-	-	-	-	-	-	-	-	0.008*
Poor progress	6	7.1	1	0.9	2	3.84	1	1.1	2	0.9	-	-	-	-	-	-	-	-	-	-	-
Cord prolapses	-	-	-	-	-	-	-	-	1	0.4	-	-	-	-	-	-	-	-	-	-	0.386*
Severe prematurity	-	-	-	-	-	-	-	-	-	-	-	-	-	-	-	-	-	-	1	1.2	0.049*
Chorioamnionitis	-	-	-	-	-	-	-	-	1	0.4	-	-	-	-	-	-	-	-	-	-	-
Others	-	-	6	5.2	9	17.30	7	7.8	7	3.1	-	-	-	-	-	-	-	-	1	1.1	< 0.001*
**Previous caesarean section**																					
PSC1 Declining VBAC	-	-	1	0.9	-	-	1	1.9	52	23.2	-	-	-	-	1	4.0	-	-	2	2.3	-
PSC1 Failed VBAC	-	-	-	-	-	-	2	2.2	19	8.5	-	-	-	-	-	-	-	-	-	-	< 0.001*
PSC1+PPROM	-	-	-	-	-	-	-	-	3	1.3	-	-	-	-	-	-	-	-	-	-	-
PSC1+Hypertension	-	-	-	-	-	-	-	-	15	6.7	-	-	-	-	-	-	-	-	2	2.3	-
PSC1+Diabetes	-	-	-	-	-	-	-	-	5	2.2	-	-	-	-	-	-	-	-	-	-	-
PSC2 in labour	-	-	-	-	-	-	-	-	34	15.4	-	-	-	-	-	-	-	-	6	7.0	-
PSC2 Elective	-	-	-	-	-	-	-	-	22	9.8	-	-	1	7.7	1	4.0	-	-	3	3.5	< 0.001*
PSC3 Elective	-	-	-	-	-	-	-	-	5	2.2	-	-	-	-	-	-	-	-	1	1.7	-
**Total**	**84**	**-**	**114**	**-**	**52**	**-**	**89**	**-**	**224**	**-**	**5**	**-**	**13**	**-**	**26**	**-**	**4**	**-**	**84**	**-**	**-**

CPD, cephalopelvic disproportion; IOL, induction of labour; AOL, augmentation of labour; HELLP, haemolysis, elevated liver enzymes, low platelets; APH, antepartum haemorrhage; PPROM, prolonged premature rupture of membranes, PCS1, one previous caesarean section; PCS2, two previous caesarean sections; PCS3, three previous caesarean sections; VBAC, vaginal delivery after caesarean section.

* , Fisher exact *p*-value.

### Foetal condition and neonatal outcomes

Of the 691 NSTs, 62.4% had pathological cardiotocography traces preoperatively, and 657 (94.5%) neonates were born with good Apgar scores. The median Apgar scores were 9 and 10 at 1 and 5 min, respectively. Low Apgar scores (< 7) were observed in 13.2% at 1 min and 5.2% at 5 min. Low birth weight was recorded in 28.0% of neonates. Neonatal admissions occurred in 11.5% of cases, because of prematurity and/or intrauterine growth restriction (IUGR) 33 (41.2%) and respiratory distress syndrome 21 (26.2%) (Table 5).

**TABLE 5:** Foetal condition before and after caesarean section (*N* = 695).

Variable	Parameter	*n*	%
Electronic foetal heart monitoring	Normal	212	30.7
	Suspicious	48	6.9
	Pathological	431	62.4
Condition post-caesarean section	Foetus status – Alive	721	97.6
	Foetus status – FSB	9	1.3
	Foetus status – MSB	7	1.0
	1-min Apgar score < 7	92	13.2
	1-min Apgar score ≥ 7	603	86.8
	5-min Apgar score < 7	36	5.2
	5-min Apgar score ≥ 7	657	94.5
Overall outcome	Discharged with mother	614	88.3
	Neonatal admission	80	11.5
Reasons for neonatal admission	IUGR and/or prematurity	33	41.2
	RDS	21	26.2
	Meconium aspiration syndrome	5	6.2
	Macrosomia	2	2.5
	Birth asphyxia	1	1.2
	Other	17	21.2

FSB, fresh stillbirth; MSB, macerated stillbirth; IUGR, intrauterine growth restriction; RDS, respiratory distress symptoms.

### Maternal outcomes and near-miss events

**FIGURE 1:**
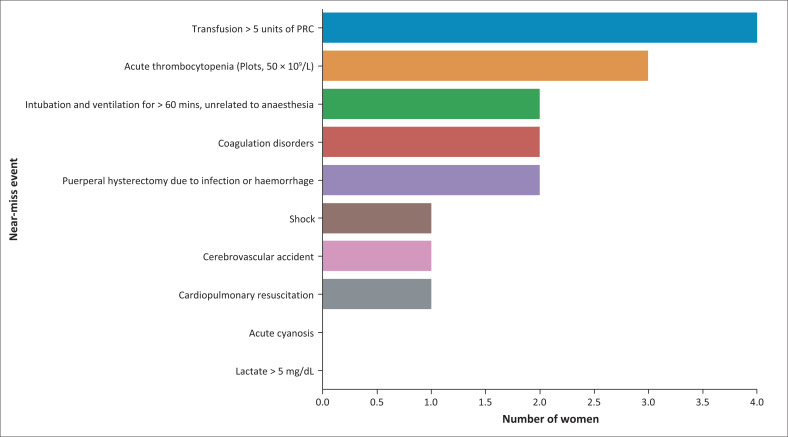
Distribution of maternal near-miss events among women undergoing caesarean section.

**FIGURE 2:**
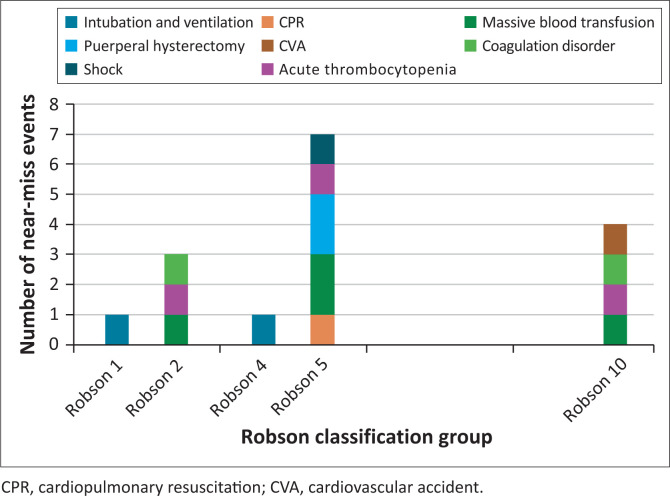
The number of maternal near-miss stratified by Robson classification group.

Most women underwent spinal anaesthesia (93.8%) and lower-segment incisions (98.8%). Caesarean section duration was less than 1 hr in 55.1% of the women. Haemostatic interventions included B-Lynch sutures (4 cases, 0.6%) and hysterectomy (2 cases, 0.4%). Postoperatively, 638 women (92.3%) were transferred to the ward, 48 (6.9%) to high care, and 2 (0.6%) to the intensive care unit (ICU). Forty-eight women (6.9%) experienced maternal complications, with 5 classified as near-miss events (0.7%). These near-miss events included massive transfusion (*n* = 4), acute thrombocytopenia (*n* = 3) and hysterectomy (*n* = 2). Most near-miss cases fell in Robson Groups 5, 2 and 10 (Figure 1 and Figure 2).

There were five maternal near-miss events:

- An unbooked woman with a prior CS, intrauterine foetal distress at 27 weeks, and ruptured uterus managed by hysterectomy.
- A multigravida with twins and acute heart failure requiring ICU admission.
- A woman with previous CS who declined repeat CS and suffered uterine rupture and neonatal birth asphyxia (Apgar 3 and 6).
- A hypertensive woman with a stillbirth resulting from placental abruption requiring ICU admission.
- A woman initially managed as an oncology case, later diagnosed with schistosomiasis, developed sepsis and septic shock post-CS.

### Association between maternal and neonatal outcomes

Adverse maternal outcomes were significantly associated with stillbirth (OR 12.10; 95% CI 4.29–34.14) and low 5-min Apgar scores (OR 5.83; 95% CI 2.79–12.18) (Table 6).

**TABLE 6:** Variables associated with the risk of adverse maternal outcome.

Variables associated with the risk of adverse maternal outcomes	OR	95% CI	*p*-value
Stillbirth	12.10	4.29–34.14	< 0.001
Low Apgar score at 5 min	5.83	2.79–12.18	< 0.001

CI, confidence interval; OR, odds ratio.

## Discussion

Over half of all deliveries during the study period (53.2%) were CS. This high CS rate reflects the significant number of high-risk pregnancies referred to a tertiary referral centre in these settings. The higher rates fall between the South African rates of 28.8% and 75% in public and private hospitals, respectively, between 2020 and 2022.^14^ It is significantly higher than the average global CS rate of 21% and Europe rate of 25.7%.^14^

Several factors contribute to the elevated CS rates in South Africa. There is a dominant belief that CS is a safer method of delivery.^15^ Additional drivers include convenience for both clinicians and patients, and concerns over medico-legal consequences when vaginal delivery outcomes are unfavourable.^14^ Furthermore, individual and socio-maternal influences, such as tocophobia (fear of childbirth), contribute significantly to CS. Tocophobia affects between 7% – 25% of nulliparous and 7.7% – 16.5% of multiparous women.^15^ While CS on maternal request is not usually permitted in state hospitals, fear of childbirth still influences decisions, particularly in women with previous caesareans.^12^

According to the Robson Classification, Group 5 (multiparous women with at least one prior CS and a singleton cephalic-term pregnancy) was the largest group contributor to the overall CS rate, accounting for 224 (32.2%) of CS cases. Women with previous CS who declined VBAC contributed 57 (8.5%) cases. These findings align with trends observed in South Africa and globally.^12^ Radaf et al. reported that Group 5 accounts for between 51.2% and 95.0% of CS in European countries, raising concerns about the ‘domino effect’ of primary CS.^13^ The Royal College of Obstetricians and Gynaecologists, however, recommends that women should be offered VBAC where appropriate.^16^

The third main contributor to the CS rate in this cohort was women with two or more previous caesareans in labour (40 cases; 5.8%). A total of 21 women (3.0%) experienced failed VBAC, and 57 (8.2%) opted for elective repeat CS. These figures suggest that encouraging VBAC could be beneficial, but further investigation into decision-making and the outcomes is required to inform the decision. The study did not capture reasons for maternal preference for repeat CS or the success rate of attempted VBACs. However, the high contribution of Group 5 underscores the importance of reducing primary CS to prevent subsequent repeat procedures.

A significant clinical driver of primary CS was foetal distress, accounting for 328 (47.2%) of total CS, which was particularly among those undergoing induction of labour (IOL), who represented 131 (18.8%) of cases. While this study did not evaluate the accuracy of foetal distress diagnosis, its frequent occurrence in Robson Groups 1, 2, 3, 4 and 10 indicates a pressing need for improved NST interpretation. Notably, Group 10 (preterm births) showed high vulnerability to foetal distress, contributing 166 (23.8%) of all cases.

Among preterm deliveries, the common contributing maternal comorbidities were obesity (52; 7.48%), hypertensive disorders (45; 6.47%), and a previous CS (21; 3.02%). Additional contributing factors included premature rupture of membranes, poor obstetric history, abruptio placentae and preterm labour. Only 14 (2.01%) preterm pregnancies were induced. Preterm deliveries were associated with poor maternal outcomes, malpresentation and poor placental decidualisation. A study conducted in the United Kingdom reported that antepartum haemorrhage (APH) complicates 3–5% of pregnancies and contributes to up to 20% of preterm births,^17^ which, in turn, elevates the risk of postpartum haemorrhage (PPH).

### Foetal outcomes

The contribution of non-stress tests (NSTs) to perinatal outcomes was also evaluated. A total of 431 (62.4%) NSTs showed pathological tracing; 657 (94.5%) neonates were born with good Apgar scores, with a mean 5-min Apgar score of 9.58; and 88.3% were discharged to the postnatal ward with their mothers. This discrepancy reflects the low positive predictive value of NSTs, approximately 50%, as previously documented.^18^ The overdiagnosis of foetal distress and misinterpretation of foetal heart rate tracings may be inflating CS rates for suspected foetal compromise. Unless more precise foetal surveillance tools become available, reducing CS rates for foetal distress will remain challenging.

Of the total CS deliveries, 168 (23.9%) were preterm. Common neonatal ICU (NICU) admissions were caused by intrauterine growth restriction (IUGR) or prematurity (33 cases; 41.2%) and respiratory distress syndrome (21 cases; 26.3%). Preterm births (< 36 weeks) were associated with lower Apgar scores and stillbirths. In 60% of cases with Apgar scores < 7 (*n* = 35; 5.03%), underlying conditions included malpresentation or maternal hypertensive disorders.

In a large cohort study in China to investigate the impact of gestational hypertension and preeclampsia on preterm birth, An et al. found that preeclampsia significantly increases the risk of preterm delivery.^19^ Velaphi et al. further found, in a CHBAH study to determine the survival rate of infants weighing 500 g – 1400 g according to birth weight and gestational age, that neonatal survival was 84% in infants weighing 1000 g – 1499 g, compared with only 32% in those < 1000 g. Key factors contributing to better outcomes included antenatal care, female sex, CS delivery, and Apgar scores > 5 at 1 or 5 min.^20^ These findings reiterate the value of quality antenatal care in preventing morbidity and mortality.

### Maternal outcomes

Most women in this cohort experienced uneventful postoperative recovery. The most common indications for referral were previous CS, preeclampsia and post-term pregnancy, consistent with expectations for a tertiary hospital. About half of the mothers (52.9%) had an ultrasound before 24 weeks of gestation, although the WHO recommends that all women receive one ultrasound before 24 weeks of gestation.^21^

In a systematic review and meta-analysis of the knowledge and utilisation level of obstetric ultrasound in African hospitals and globally, Gashaw et al. found that overall knowledge about obstetric ultrasound among mothers was 74.3%, with a pooled proportion of utilisation of 63.3% (95% CI 51.59% – 75.02%), and knowledge was identified as the key factor significantly associated with ultrasound utilisation.^22^

The most frequent postoperative complication was postpartum haemorrhage, observed in 39 cases (5.7%). Surgical interventions included haemostatic sutures (9 cases; 50%), B-Lynch sutures (4 cases; 22.2%), and hysterectomy (2 cases; 16.7%). Both hysterectomies were for placenta praevia, with one case undiagnosed antenatally. Average blood loss in these cases was 3500 mL.

Only one near miss was deemed avoidable; three involved unbooked patients, reflecting the challenges faced in managing walk-ins without prior antenatal care. Common features of these near-miss cases included massive transfusion (4 cases; 100%), thrombocytopenia (3 cases; 75%), hysterectomy (2 cases; 50%) and ventilation (2 cases; 50%).

Group 5 (previous CS) contributed to 43.7% of all near-miss cases, highlighting the increased maternal risks associated with repeat caesarean delivery.

### Correlation between maternal and neonatal outcomes

There was a strong association between maternal complications and adverse neonatal outcomes. Women with severe maternal morbidity had a 12-fold increased risk of stillbirth (OR 12.10; 95% CI: 4.29–34.14; *p* < 0.001) and a 6-fold higher risk of a low 5-min Apgar score (OR 5.83; 95% CI: 2.79–12.18; *p* < 0.001). In a prospective observational study across 22 African countries, Bishop *et al* found that maternal mortality after CS is 50 times higher than in high-income countries and is driven by peripartum haemorrhage and anaesthetic complications, with neonatal mortality double the global average. This underscores the need for early detection of maternal complications, appropriate perinatal planning and rapid perinatal interventions to improve outcomes.^23^

### Strengths and limitations

This study contributes significantly to the classification of risk factors associated with CS. It further highlights the obstetric challenges of a high-risk tertiary facility in a low-income country because of a high influx of high-risk mothers and complicated labour. One could reasonably argue that the CS rates at CHBAH might be significantly reduced through more meticulous interpretation of pathological non-stress tests (NSTs), particularly given that such findings accounted for nearly half of all caesarean deliveries.

Because of cross-sectional sampling, variation in diseases and indications for CSs cannot be ignored. It also does not account for the influence of the patient’s decision-making process and autonomy. Although single-centred, the number and referral pathway to CHBAH make it representative of the situation in the province and beyond.

### Recommendations

Healthcare practitioners should be counselled on how to appropriately select mothers for an NST and how to interpret an NST.

How can interpretation be improved?

Further studies need to be done to reduce primary or repeat CS or looking at the average parity of patients with repeat CS, and the use of contraception post first CS.

Further research is recommended on the accuracy of the means of diagnosing foetal distress, as most foetuses had a favourable outcome.

## Conclusion

This study identified a high CS rate of 53.2% at a tertiary institution, driven by high-risk referrals, repeat caesarean deliveries and indications such as foetal distress. Although VBAC remains a safe alternative for many women, its uptake and success remain low, with only 3% having a failed VBAC, and 8.3% opting for elective repeat procedures. Foetal distress, particularly in cases with abnormal intrapartum monitoring, was the most frequent indication for emergency CS, highlighting the need for improved interpretation of cardiotocographs and timely clinical decision-making. Given the substantial burden of repeat CSs and their contribution to maternal near-miss events, targeted interventions are needed. These include enhancing antenatal risk stratification, offering external cephalic version for breech presentations starting at 36 weeks, and promoting evidence-based labour management. Strengthening patient education and preconception counselling, especially in women with pre-existing maternal diseases and chronic conditions, may help reduce unnecessary caesarean deliveries and improve both maternal and neonatal outcomes in tertiary care settings in South Africa.

## References

[1] Tollånes MC. Increased rate of caesarean sections – Causes and consequences. Tidsskr Nor Laegeforen. 2009;129(13):1329–1331. 10.4045/tidsskr.08.0453 19561658

[2] Niekerk AV, Soma-Pillay P. Caesarean births: South Africa’s rates are too high – They can be dangerous for mothers and babies. The Conversation [serial online]. 2024 [cited 2025 Jul 27]. Available from: http://theconversation.com/caesarean-births-south-africas-rates-are-too-high-they-can-be-dangerous-for-mothers-and-babies-226516

[3] Solanki GC, Cornell JE, Daviaud E, Fawcus S. Caesarean section rates in South Africa: A case study of the health systems challenges for the proposed National Health Insurance. S Afr Med J. 2020;110(8):747. 10.7196/SAMJ.2020.v110i8.14699 32880299

[4] Antoine C, Young BK. Caesarean section one hundred years 1920–2020: The good, the bad and the ugly. J Perinat Med. 2021;49(1):5–16. 10.1515/jpm-2020-0305 32887190

[5] NCCEMD. National committee for confidential enquiry into maternal deaths. Saving mothers 2017–2019 annual report on confidential inquiries into maternal deaths; in South Africa: Comprehensive report [homepage on the Internet]. 2020. Available from: https://www.health.gov.za/wp-content/uploads/2023/11/Saving-Mothers-2017-2019-Technical-Report-FINAL-2-1.pdf

[6] NCCEMD. National committee for confidential enquiry into maternal deaths. Saving mothers 2023 annual report on confidential enquiries into maternal deaths; in South Africa: Comprehensive report [homepage on the Internet]. 2023. Available from: https://knowledgehub.health.gov.za/system/files/2024-02/Executive%20%20Report%20%202020-2022%20Presentation%20Dr%20Sylvia%20Cebekulu_.pdf

[7] Soma-Pillay P, Pattinson RC, Langa–Mlambo L, Nkosi BSS, Macdonald AP. Maternal near miss and maternal death in the Pretoria Academic Complex, South Africa: A population-based study. S Afr Med J = Suid-Afrikaanse Tydskrif Vir Geneeskunde. 2015;105(7):578–563. 10.7196/SAMJnew.8038 26428756

[8] Guidozzi DF, Branch S, Chauke L. Maternal and fetal outcomes following delivery in a tertiary hospital in Johannesburg, South Africa. S Afr J Obstet Gynaecol. 2019;24(3):74. 10.7196/sajog.1396

[9] Sobhy S, Arroyo-Manzano D, Murugesu N, et al. Maternal and perinatal mortality and complications associated with caesarean section in low-income and middle-income countries: A systematic review and meta-analysis. Lancet. 2019;393(10184):1973–1982. 10.1016/S0140-6736(18)32386-9 30929893

[10] WHO. Robson classification: Implementation manual [homepage on the Internet]. 2017 [cited 2024 Nov 25]. Available from: https://www.who.int/publications/i/item/9789241513197

[11] Tiwari M, Singh S, Kushwah BS. Robson’s ten group classification: a tool for predicting caesarean section rates. Int J Reprod Contracept Obstet Gynecol. 2023;12(3):595–602.

[12] FD Rubgega, Soma-Pillay P, Becker P. Caesarean section indications and outcomes at a tertiary level hospital in South Africa. Obstet Gynaecol Forum [serial online]. 2023 [cited 2023 Feb 10];31(3):8–11. Available from: https://www.ajol.info/index.php/ogf/article/view/241391

[13] Radaf VE, Campos LN, Savona-Ventura C, Mahmood T, Zaigham M. Robson ten group classification system for caesarean sections across Europe: A systematic review and meta-analysis. Eur J Obstet Gynaecol Reprod Biol. 2025;305:178–198. 10.1016/j.ejogrb.2024.11.052 39705988

[14] Betrán A, Torloni MR, Zhang JJ, et al. WHO statement on caesarean section rates. BJOG: Int J Obstet Gynaecol. 2015;123(5):667–670. 10.1111/1471-0528.13526 PMC5034743 26681211

[15] Pavlidou E, Antasouras G, Papadopoulou SK, et al. Association of maternal risk factors with the prevalence of caesarean section deliveries: A cross-sectional study. Med Sci. 2023;11(4):66. 10.3390/medsci11040066 PMC10594507 37873751

[16] RCOG. Birth after previous caesarean birth: Green-top guideline No. 45 [homepage on the Internet]. 2015 [cited 2025 Sep 04]; p. 1–31. Available from: https://www.rcog.org.uk/guidance/browse-all-guidance/green-top-guidelines/birth-after-previous-caesarean-birth-green-top-guideline-no-45/

[17] Varouxaki N, Gnanasambanthan S, Datta S, et al. Antepartum haemorrhage. Obstet Gynaecol Reprod Med. 2018;28(8):237–242. 10.1016/j.ogrm.2018.07.001

[18] Dound NC, Pajai S, Singh A. The correlation of intraoperative findings and foetal outcome in cases taken for caesarean section based on non-reassuring cardiotocographic changes – A review article. J Fam Med Prim Care. 2022;11(10):5894–5898. 10.4103/jfmpc.jfmpc_440_22 PMC9810911 36618251

[19] An H, Jin M, Li Z, et al. Impact of gestational hypertension and pre-eclampsia on preterm birth in China: A large prospective cohort study. BMJ Open. 2022;12(9):e058068. 10.1136/bmjopen-2021-058068 PMC9516080 36167382

[20] Velaphi SC, Mokhachane M, Mphahlele RM, Beckh-Arnold E, Kuwanda ML, Cooper PA. Survival of very-low-birth-weight infants according to birth weight and gestational age in a public hospital: original article. S Afr Med J. 2005;95(7):504–509. 16156449

[21] World Health Organization (WHO). WHO recommendations on antenatal care for a positive pregnancy experience: Summary. Geneva: WHO; 2018. 28079998

[22] Gashaw A, Figa Z, Abebe Z, Demeke AD, Sime Y. Knowledge and utilization of obstetric ultrasound and associated factors among pregnant mother in Africa: A systematic review and meta-analysis. Ultrasound J. 2025;17(1):1–14. 10.1186/s13089-025-00420-w 40029487 PMC11876473

[23] Bishop D, Dyer RA, Maswime S, et al. Maternal and neonatal outcomes after caesarean delivery in the African surgical outcomes study: A 7-day prospective observational cohort study. Lancet Glob Health. 2019;7(4):e513–e522. 10.1016/S2214-109X(19)30036-1 30879511

